# Performance, Accuracy and Generalization Capability of RFID Tags’ Constellation for Indoor Localization

**DOI:** 10.3390/s20154100

**Published:** 2020-07-23

**Authors:** Elias Hatem, Sara Abou-Chakra, Elizabeth Colin, Jean-Marc Laheurte, Bachar El-Hassan

**Affiliations:** 1 School of Engineering, EFREI Paris, 94800 Villejuif, France; elizabeth.colin@efrei.fr; 2 Faculty of Technology, Lebanese University, Aabey 24375, Lebanon; sabouchakra@ul.edu.lb (S.A.-C.); bachar_elhassan@ul.edu.lb (B.E.-H.); 3 Electronics, Communication Systems and Microsystems Laboratory (ESYCOM), Paris Est University, 77420 Champs-sur-Marne, France; jean-marc.laheurte@u-pem.fr

**Keywords:** indoor localization, received signal strength indicator, multiple input and single output, UHF RFID tags, constellation of tags, position error

## Abstract

Indoor localization has recently witnessed an increase in interest due to its wide range of potential services. Further, the location information is very important in many applications, such as the Internet of Things, logistics, library management and so on. Hence, different technologies and techniques have been proposed in the literature for indoor localization systems. Most of these systems present the disadvantages of a poor performance, low accuracy and high cost. However, thanks to its low cost, high accuracy and non-line-of-sight detection, radio frequency identification (RFID)-based localization has increasingly become the most used technology for indoor localization. In this paper, we propose an innovative approach based on the multiple input single output (MISO) protocol to improve the accuracy of a low-cost RFID localization system. Whereas most traditional systems use a single tag for localization, the proposed architecture encourages the use of a group of RFID tags named as a constellation. According to experimental results and based on the signals’ diversity, the location accuracy is improved to get an estimated position error of 81 cm at the cumulative distribution function of 90%.

## 1. Introduction

Indoor positioning systems are used for various location-based applications. Many researches about radio frequency identification (RFID)-based localization have been proposed for its easy deployment and successful utilization in harsh environments [1,2,3]. It is rapidly being adopted as one of the most affordable and scalable technologies of wireless identification, tracking and detection [4]. Different localization techniques are used, such as the time of arrival (ToA) [5,6], time difference of arrival (TDoA) [7,8] or received signal strength (RSS) [9,10] to locate the wireless terminal. 

Recently, several methods based on the RSS have been proposed in the literature [11,12,13]. The main reason for using the received signal strength is the availability of this data in smart technologies and particularly in RFID. However, RSS measurements in indoor applications are affected by multipath due to the reflection or diffraction from walls or surrounding objects and the shadowing of the line-of-sight propagation path by intervening obstacles [14]. These propagation phenomena affect the channel stability and the location accuracy obtained by the RSS localization technique.

Localization based on RFID technology can be categorized into two types: reader and tag localization. In the case of reader-based localization, the RFID reader is usually attached to the tracked person or object while tags are installed in the environment. Such fixed tags can be active or passive. This localization technique has the advantage of providing the absolute location of the moving object, and also the displacement information associated to the movement. The solution presented in [15] used thirty fixed RFID tags with several antenna readers. After applying Bayes classifier, the obtained location accuracy based on the RSS is 1.2 m in a corridor with an area of 45 m^2^. In contrast, tag-based localization is suitable for many applications such as locating goods in warehouses or tracking luggage in airports. It can provide the same location accuracy as that obtained with the reader-based approach. For instance, [16] shows a novel hybrid system for indoor localization; two algorithms were introduced: SA-LANDMARC and COCKTAIL, in a tested area of 36 m^2^. The accuracy reached 70 and 45 cm, respectively. Despite SA-LANDMARC’s implementation simplicity and COCKTAIL’s accuracy, the achieved high precision, using both algorithms, is due to the dense deployment of RFID tags. In short, as the reader or tag-based indoor localization systems present almost the same location accuracy, we will propose a reader-based approach aiming to avoid the dense deployment of RFID tags.

In this context, the received signal strength (RSS) stability is an important parameter to be considered. Through the signal diversity, this stability could be achieved [17]. Furthermore, the multiple antennas technology was proven to be efficient by improving the spectral efficiency over that of conventional single antenna systems [18,19,20]. The capacity limits of the multiple antennas technology were extensively studied in various practical scattering propagation environments [21,22,23]. Besides, the spatial diversity of the emitted signals is well represented by multiple input single output (MISO) systems. These systems introduced many techniques to reduce the effect of multipath fading [19,20,21]. In addition, the classical approaches consist of using multiple receiving antennas and performing combining or selection and switching techniques to improve the quality of the received signal. Based on this idea, the concept of using multiple antennas for localization is very challenging. Therefore, our proposed approach is focused on using a group of tags that work together at the same frequency in a reader-based localization system. We named this configuration a constellation of tags. Several techniques can be used to exploit the antennas signals’ diversity. In this study, the average of the received signals is suggested and assessed.

This work contributes to the performance an accurate indoor positioning system via the constellation of RFID tags. This approach aims to improve the reliability of the RSS information by the signals’ diversity. The constellation’s shape and dimensions will be the main parameters that should be examined. Experimental results for position errors will be assessed for two scenarios: single emitting tags and the constellation of emitting tags in a reader-based localization system. Finally, the novel approach of the constellation of tags is validated by achieving high estimation accuracies.

The rest of the paper is organized in five sections. The RFID reader-based localization system is introduced in Section 2. Section 3 expands the proposed constellation approach. Section 4 compares the performance of the constellation of tags against those obtained with a single tag in terms of distance and position estimations. Finally, the last section focuses on conclusions based on the obtained results.

## 2. RFID-Based Localization System

In this section, the proposed RFID reader-based localization system is introduced as a baseline. Then, the propagation model [24] applied in the following sections is presented. Further, the estimation of the tag-reader distance is derived from the received power (RSS). Finally, positioning based on the multilateration technique is presented in the last part of this section.

### 2.1. Localization System Principle

As already stated, RFID localization systems can be classified into two types, i.e., reader localization and tag localization. In the case of reader localization, the RFID active tag sends electromagnetic signals to the reader, which in turn measures the received signal strength indicator (RSSI). The proposed system is considered as a reader-based localization system where the position of the RFID reader, in an observation area, is estimated. The used RFID tags operate at the Ultra High Frequency (UHF) frequency of 433 MHz as this frequency presents a larger communication range and is less affected by multipath fading [25].

**Figure 1:**
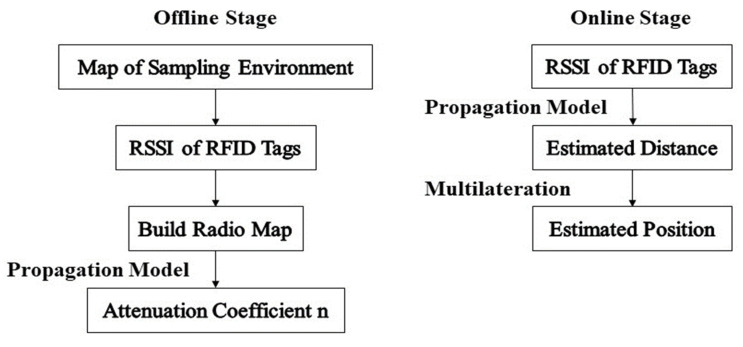
Block diagram of the offline and online stages.

The main concept of our localization method can be divided on two stages: the offline and online stage, as presented in Figure 1. The offline stage represents a calibration phase for the environment and the online stage is the positioning phase. During the offline stage, the received signal strength is collected at sampling locations to build a radio map of the indoor environment. By applying the considered propagation model, the environment attenuation coefficient is extracted. Then, in the online stage, the tag-reader distance is estimated using the propagation models. Finally, the estimated distances are used to determine the reader position via the multilateration technique.

### 2.2. Propagation Models

Knowledge of the signal paths can greatly improve an indoor wireless localization system’s performance. Hence, numerous experimental and theoretical studies regarding indoor propagation models can be found in the literature [26,27,28].

In a previous publication of the authors [24], two new empirical indoor propagation models were introduced. The first one is the dual one slope propagation model (DOSM) defined as follows:

(1) $$PL\left(d\right)=\{\begin{array}{c} PL_{0}+10.n_{1}.log_{10}\left(d\right)+X_{1}\;\;\;\;\;\;d\leq d_{1}=3\lambda \\ PL_{0}+10.n_{2}.log_{10}\left(d\right)+X_{2}\;\;\;\;\;\;d>d_{1}=3\lambda \end{array}$$

The second one is the dual one slope with second order propagation model (DOSSOM), expressed by:

(2) $$PL\left(d\right)=\{\begin{array}{c} PL_{0}+10.n_{1}.log_{10}\left(d\right)+X_{1}\;\;\;\;\;\;d\leq d_{1}=3\lambda \\ a.\log_{10}{\left(d\right)}^{2}+b.log_{10}\left(d\right)+c\;\;d>d_{1}=3\lambda \end{array}$$

In Equations (1) and (2), PL(d) is the received power in dBm at the distance *d* in meters, $PL_{0}$ is the free space path loss at the distance of 1 m, $n_{i}$ is the path loss exponent corresponding to the $i^{th}$ path and $X_{i}$ is a lognormal variable with standard deviation throughout the $i^{th}$ path. 

In Equation (2), *a*, *b* and *c* are the constant parameters of the second order polynomial model. They are determined by solving a system of three unknowns that can be setup by considering three pairs of particular values of *PL* and *d*.

### 2.3. (Tag-Reader) Distance Estimation

**Figure 2:**
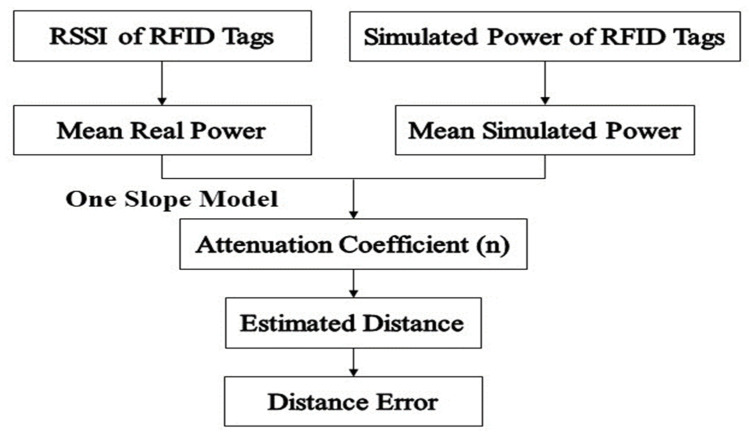
Steps for the estimated distance error calculation.

The reader-tag distance can be estimated with any state of the art indoor propagation models. In this section, the estimate is derived from the one slope model as a reference since it is the most common one, but the later models presented previously are applied. Figure 2 represents a block diagram of the distance error calculation. The received power $(\hat{P_{\mathrm{dBm}})}$ is given by:

(3) $$\hat{P_{\mathrm{dBm}}}=-10n(\log_{10}d)+P_{0}$$

Then, the distance can be estimated as:

(4) $$\hat{d}=10^{\left(\;\frac{\hat{P_{\mathrm{dBm}}}-P_{0}}{-10n}\right)}$$

$P_{0}$ is the received signal strength at a distance of one meter; n is the channel propagation constant, which is also referred to as the path loss exponent.

Finally, the distance error ($\epsilon_{d})$ is calculated as follows:

(5) $$\epsilon_{d}=|d_{r}-\hat{d}|$$

where $d_{r}$ is the real tag-reader distance.

### 2.4. The Multilateration Technique

**Figure 3:**
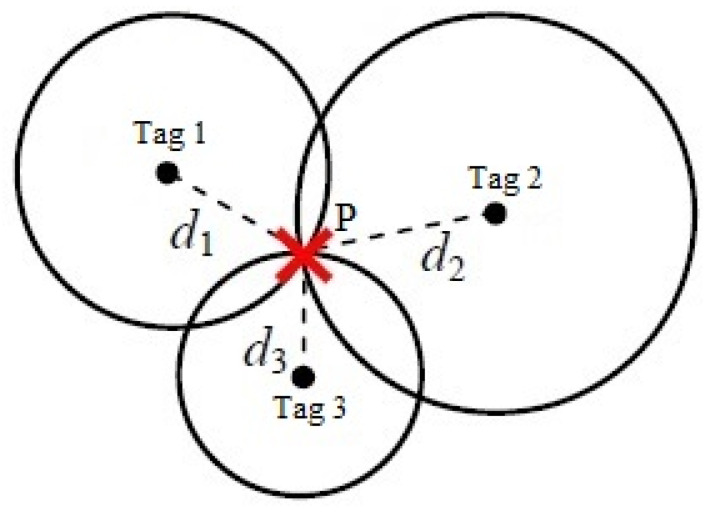
Trilateration technique-based positioning.

Many indoor positioning systems are based on the trilateration approach. The term trilateration refers to the process that estimates the target position based on the three estimated distances [29]. The estimated distance based on the RSS is represented by a circle with a radius around the fixed tags. The intersection of the three circles provides a common point or coverage area of the received signals (Figure 3). However, in most cases, each radius is equal to the distance between the RFID tag and the moving reader. To mitigate the radius variation, numbers that are proportional to the radius of each circle are temporarily added to each distance in order to lengthen the radii of the circles. Then, the estimated location includes error due to the radius extension. Thus, some recent advanced algorithms have been suggested to improve the positioning accuracy [6,8]. 

The multilateration technique has the same principle as that of trilateration but it requires more than three fixed transmitted tags to locate an object. It is applied to increase the location accuracy. In this context, Reference [8] implements the multilateration technique to overcome the audio receiving latency problem. In our study, the accuracy will be improved by introducing the constellation approach that will be presented in the next section. Moreover, we are working in a two-dimensional plane; the RFID tag and the reader are at the same height. Thus, the unknown applicate parameter can be omitted. For instance, P(a,b) represents the unknown reader’s location. ($x_{i}$,$y_{i}$) represents the $i^{th}$ known tag coordinates, and $d_{i}$ represents the distance between the reader and the $i^{th}$ tag. According to the Euclidean distance formula, nonlinear equations are formulated as follows:

(6) $$\begin{array}{c} {\left(a-x_{1}\right)}^{2}+{\left(b-y_{1}\right)}^{2}=d_{1}{}^{2}\\ {\left(a-x_{2}\right)}^{2}+{\left(b-y_{2}\right)}^{2}=d_{2}{}^{2}\\ {\left(a-x_{3}\right)}^{2}+{\left(b-y_{3}\right)}^{2}=d_{3}{}^{2}\\ {\left(a-x_{4}\right)}^{2}+{\left(b-y_{4}\right)}^{2}=d_{4}{}^{2} \end{array}$$

By expanding and regrouping the terms in Equation (6) we obtain:

(7) $$A.\left(\begin{array}{c} a\\ b \end{array}\right)=B$$

With

(8) $$A=2\left(\begin{array}{cc} \left(x_{2}-x_{1}\right) & \left(y_{2}-y_{1}\right)\\ \left(x_{3}-x_{1}\right) & \left(y_{3}-y_{1}\right)\\ \left(x_{4}-x_{1}\right) & \left(y_{4}-y_{1}\right) \end{array}\right)$$

And

(9) $$B=\left(\begin{array}{c} d_{1}^{2}-d_{2}^{2}-[(x_{1}^{2}-x_{2}^{2})-(y_{1}^{2}-y_{2}^{2})]\\ d_{1}^{2}-d_{3}^{2}-\left[(x_{1}^{2}-x_{3}^{2}\right)-(y_{1}^{2}-y_{3}^{2})]\\ d_{1}^{2}-d_{4}^{2}-[(x_{1}^{2}-x_{4}^{2})-(y_{1}^{2}-y_{4}^{2})] \end{array}\right)$$

The solution that corresponds to the intersection of the circles and determines the reader’s coordinates is obtained by inverting matrix A in Equation (8) as follows:

(10) $$\left(\begin{array}{c} a\\ b \end{array}\right)\;=A^{-1}B$$

## 3. The Constellation Approach

In indoor localization, the main objective of introducing the concept of the constellation of RFID tags is to minimize the location uncertainty. The constellation is a group of tags that work at the same frequency and close to each other. In this section, a MISO-based indoor localization system is introduced. Then, the constellation approach is elaborated with different radii, shapes and number of tags. By applying the one slope propagation model, the mean estimated distance errors of these constellations are analyzed and compared to each other to reach the optimal constellation of tags. 

### 3.1. MISO-Based Indoor Localization System

The MISO architecture is one of the several forms of smart antenna technologies with only one receiving antenna. The performance of wireless communication systems can be improved by adopting multiple antennas at the transmitting side [19]. While deeply looking in the literature, few papers present works using MISO for localization purposes. Most traditional researches are focused on the channel capacity [30,31]. In addition, References [32,33] reported that MISO systems bring more signal diversity than single input single output (SISO) systems. Notwithstanding these previous studies, Reference [34] proposes a comparative study of RSSI-based localization algorithms for wireless sensor networks (WSNs) in indoor environments using spatial diversity. They elaborate that the single input multiple output (SIMO) and MISO systems present similar performances. The obtained results show that the usage of a multiple antennas system significantly improves the localization accuracy to 87 cm with fourteen receiving antennas in an indoor environment of 400 m^2^. Additionally, Reference [35] proposes an indoor localization system based on a single-frequency continuous-wave Doppler radar sensor. It was implemented under the form of SIMO architecture with redundant receiving channels. The method was validated by simulations without any additional noise. Two different trajectories were recovered in a simulated environment scenario of 0.8 × 0.8 m^2^. The accuracy error, between the optimized and the actual trajectories, was 11.4 cm and 14.1 cm, respectively. However, Reference [34] implemented a WSN in a homogenous indoor environment and Reference [35] validated their approach by simulations only and without any interference. In the following, we present an innovative technique to improve the accuracy of a low-cost RFID localization system in a real complex classroom environment.

### 3.2. Constellation of UHF-RFID Tags for Reader Localization

**Figure 4:**
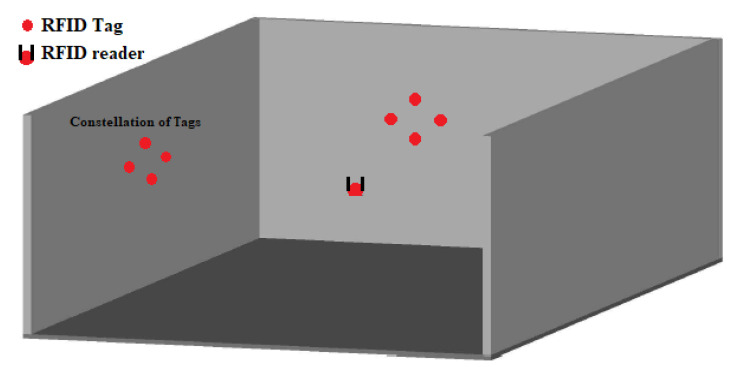
Constellation of four Radio Frequency IDentification (RFID) tags.

The RFID reader-based localization system consists of tags, a reader and the acquisition of the RSS values. The suggested constellation of tags can have different shapes and may include a variable number of tags. For instance, Figure 4 presents the constellation of four RFID tags for reader localization. The work is focused on investigating the optimal constellation’s radius, shape and the optimal number of tags constituting it. As multiple transmit antennas can reduce multipath by benefiting from the signals’ diversity, different experiments with the constellation of RFID tags will be well analyzed, in the following sections, with the aim of increasing the location accuracy.

### 3.3. Optimal Constellation’s Radius 

**Figure 5:**
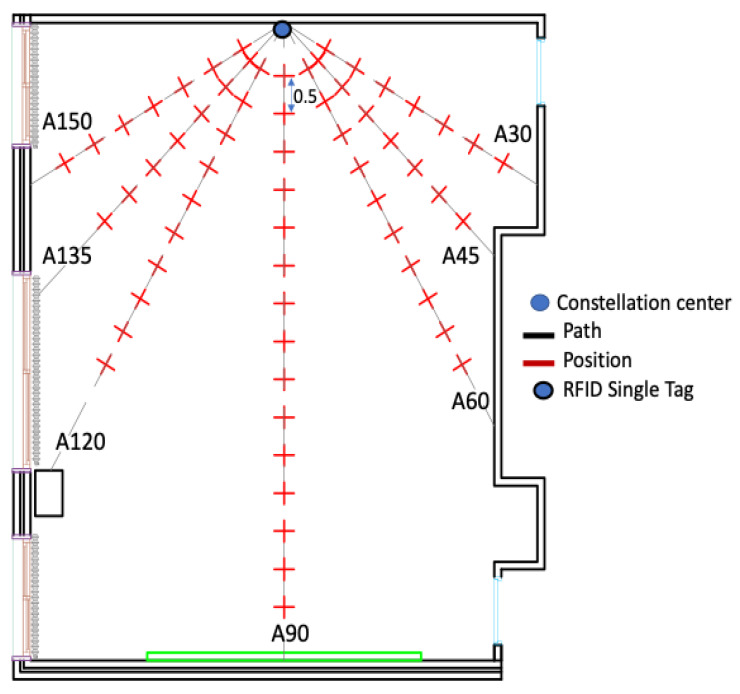
Two-dimensional layout of the classroom environment.

**Figure 6:**
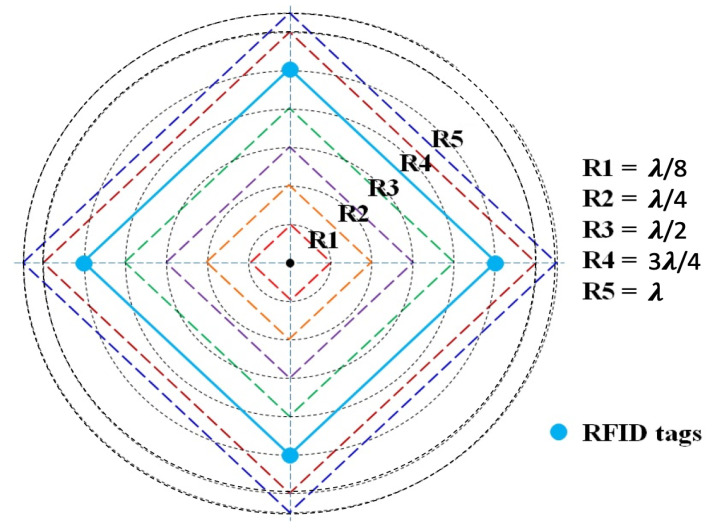
Different radii for the constellation of tags.

In this subsection, different radii of the constellation are empirically evaluated [36]. The center of the constellation was on the center of the front wall. The RFID reader was moved forward with a step equal to 50 cm to collect the received signal strength at each position as presented in Figure 5. Signals emitted by the group of tags and captured by the reader antenna were combined by the averaging technique. Furthermore, to determine the optimal radius of the constellation of RFID tags, the estimated tag-reader distance errors were analyzed for different radii starting from *λ*/8 up to *λ* (Figure 6).

Table 1 shows the mean estimated distance errors over the track A90 (Figure 5) and the different constellation radii.

Based on the collected data, it can be noticed that the mean estimated distance error over track A90 presents a minimal error for the radius equal to *λ*, whereas the maximum mean distance error was obtained while the radius of the constellation was equal to *λ*/2. Moreover, the standard deviation presents almost less variation with the radius equal to *λ*. Thus, a constellation of tags with radius *λ* was examined in the following tests.

### 3.4. Optimal Shape and Number of Tags 

**Figure 7:**
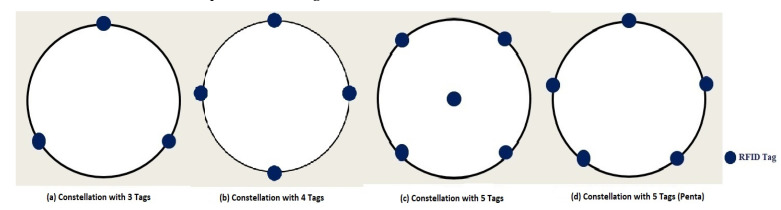
Constellation of tags with different number of tags.

In order to determine the best constellation features and after the selection of the optimal radius, it is also challenging to determine the best shape and the optimal number of tags that constitute the constellation. Several shapes, with radii equal to *λ*, are studied: a triangle constellation with three tags (Figure 7a), diamond shape with four tags (Figure 7b), square cross with five tags (Figure 7c) and pentagon with five tags (Figure 7d). Table 2 shows the mean estimated distance errors for the different shapes and the tag number of the constellation.

It is apparent that there are fewer errors in the constellation with five tags where one of the tags is at the constellation center (Figure 7c). However, adding an extra tag to the four tag constellation, in order to improve the accuracy by just 2.5 cm, makes the choice of a four tag constellation (diamond shape) more reasonable.

## 4. Constellation vs. Single Tag

In this section, the RFID equipment, the classroom where measurements and simulations were conducted, as well as a brief description of the different scenarios are presented. Then, the performances of the optimal constellation of tags are compared to those obtained with the single tag under the same conditions. Finally, by applying the multilateration technique, the location accuracy with the four tag constellation and with the single tag are presented and compared to show the improvements obtained with the constellation configuration.

### 4.1. RFID Equipment

**Figure 8:**
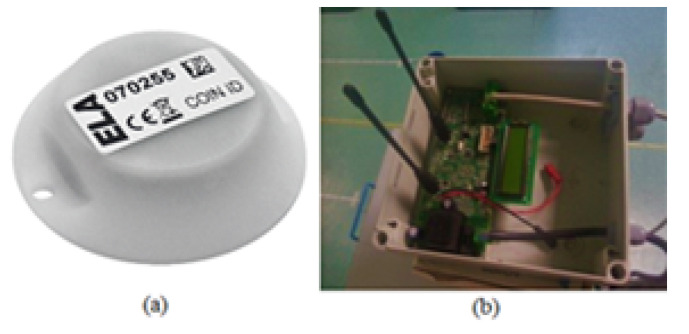
(**a**) RFID tag and (**b**) RFID reader.

To determine the constellation of tags’ performance in a classroom environment with the multilateration technique, sixteen (four constellations) RFID active tags, operating at 433 MHz, from Ela-Innovation were used. Four RFID tags were installed in the single tag localization scenario. In addition, one RFID reader was localized [37]. Figure 8a,b show the RFID tag and reader, respectively.

### 4.2. Experiment Environment

**Figure 9:**
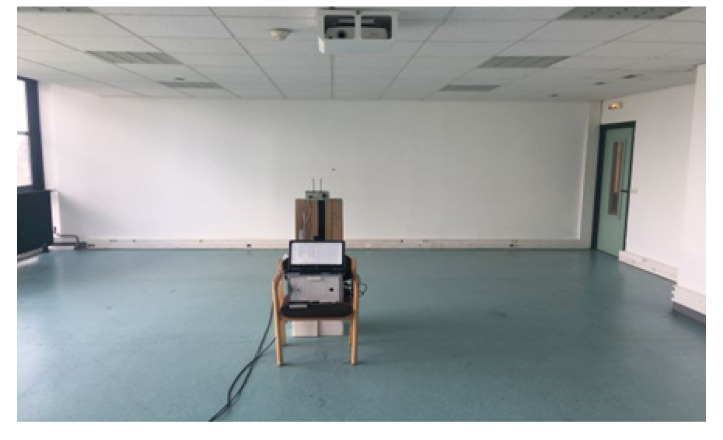
Indoor site of experiments in a classroom.

The experiment was conducted in a classroom on the fourth floor of Efrei-Paris Engineering School. It is a typical classroom with an area of approximately 63.75 m^2^. This unfurnished environment is still complex because it is occupied by some unmovable objects such as: a heater, LCD projector, fire detector and speakers. The classroom is illustrated in Figure 9. 

**Figure 10:**
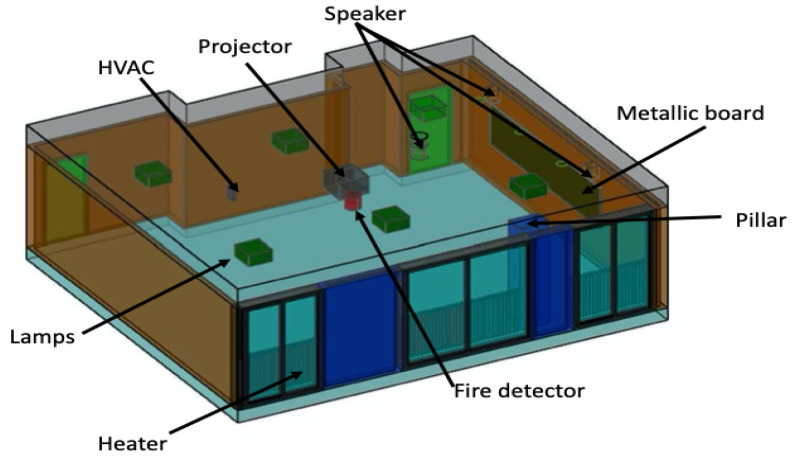
Three-dimensional layout of the classroom.

Furthermore, in order to validate the constellation approach while avoiding the long durations needed for on-site measurements, a reliable modelling of the classroom environment can be a suitable alternative, as shown in Figure 10 [38]. The simulation is performed using a WinProp tool [39] and is based on 3D ray-tracing. The behavior of the constellation of tags was studied in-depth over track A90 (Figure 5) in Section 3. In the following, the mean estimated distance error of the optimal constellation of tags is compared to that obtained with the single tag within the same scenario. Then, we end by presenting the estimated position errors over all the classroom environment.

### 4.3. Evaluation of Distances Estimation

To evaluate the optimal constellation of tags’ performance (diamond shape with a radius equal to *λ*), a comparison with the single tag configuration is essential. The single tag was fixed and, alternatively, the center of the constellation was located on the center of the front wall as shown in Figure 5. As discussed in Section 3, the MISO system presents more signal diversity compared to the SISO system. The received power values at various positions were measured to characterize the signal behavior in terms of the tag-reader distance over tracks A60, A90 and A120 (Figure 5). These tracks presented a greater number of positions when compared to other tracks. Over each track, the estimated distance errors were analyzed first while a single tag is used, then with the optimal constellation of tags and then with each tag of the optimal constellation independently to present the behavior of each one. The importance of presenting their behaviors is to prove that each tag’s signal takes a different path to reach the reader due to the multipath effects in indoor environments. Moreover, the comparison is realized by data collected either by measurements and by simulations following the block diagram illustrated in Figure 2.

#### 4.3.1. Measurements Accuracy

Based on measurements, the estimated distance errors for the single tag and the constellation of tags scenarios will be evaluated. They are determined over the three tracks: A60, A90 and A120. Table 3 shows the measured tag-reader mean estimated distance errors (MDEs). 

Based on the MDEs presented in Table 3, it can be noticed that the constellation of four tags presents fewer errors compared to single tag’s results. In addition, the constellation of tags also presents more stability as the standard deviation is smaller than that for the single tag system. Thus, the concept of using multiple transmitting antennas, to reduce the effect of multipath fading on the distance estimation, is well validated. 

**Figure 11:**
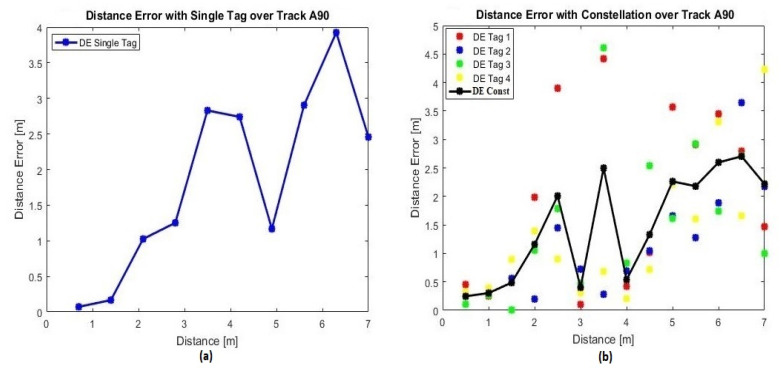
Estimated distance errors for single tag and constellation of tags scenarios over track A90.

Furthermore, Table 3 presents a sample of the estimated distance errors’ distribution over track A90 for: the respective scenarios for single tags (Figure 11a), optimal constellation of tags and each tag of the optimal constellation independently (Figure 11b). As discussed in Section 3, MISO systems achieve a better performance in terms of reliability through signal diversity [31,32]. Referring to Figure 11b, the estimated distance error, at each position, differs from one tag to another. Thus, combining multiple signals emitted by a group of tags in a constellation, via the averaging technique to exploit signal diversity, presents very promising results.

#### 4.3.2. Simulations

The whole experiment and procedure were reproduced in the simulated environment. Table 4 illustrates the mean estimated distance errors for the system with a single tag and with the constellation of tags. In short, it is obvious that the constellation performs better than single tags thanks to signal diversity. Moreover, it can be noticed that measurements and simulations present very similar results.

#### 4.4. Radio Map

**Figure 12:**
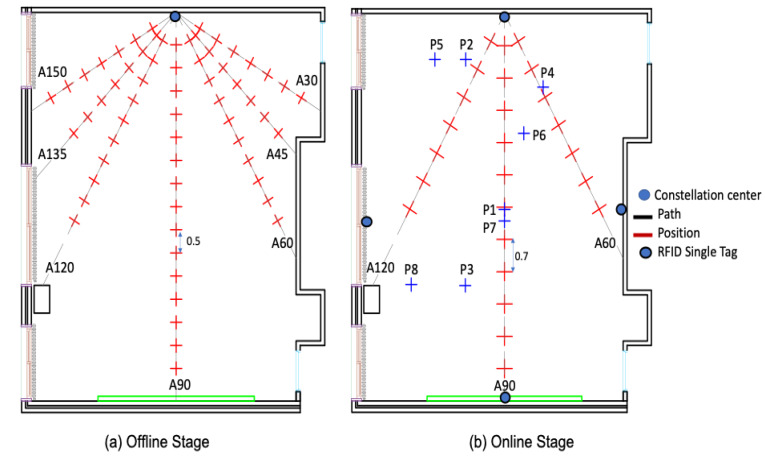
Two-dimensional layout of the classroom environment (offline and online stages).

As already stated, the experiment was performed in an unfurnished classroom environment where the signal propagation is characterized over seven paths, A30 through A150 (Figure 12a). Thus, for the calibration, one RFID tag is placed on the center of the front wall as shown in Figure 12a. In the offline stage, the received signal strength is collected every 50 cm over the seven trajectories. The propagation models, described in Section 2, were applied over each track to calculate the corresponding attenuation coefficient (Figure 12a). 

To assess the effectiveness of the classroom calibration in the offline stage, another distance between every two adjacent positions was considered for the online stage. Thirty-two RSS values from different locations were collected. Only 24 locations are uniformly distributed in the space with a distance of 70 cm over the three paths, A60, A90 and A120, as illustrated in Figure 12b. Eight other positions are chosen randomly to evaluate the localization accuracy.

#### 4.5. Assessment of the Positioning Performance

**Figure 13:**
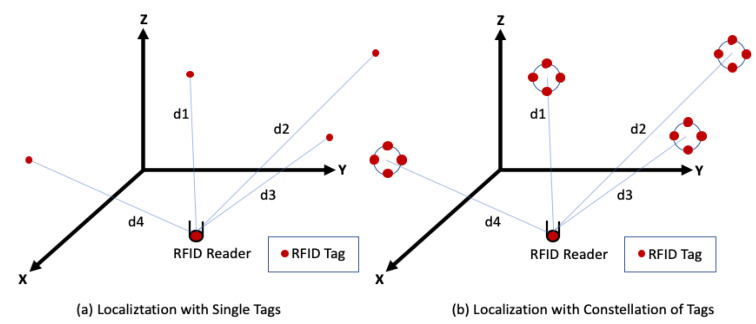
Multilateration technique applied for both the constellation of tags and single tag scenarios.

**Figure 14:**
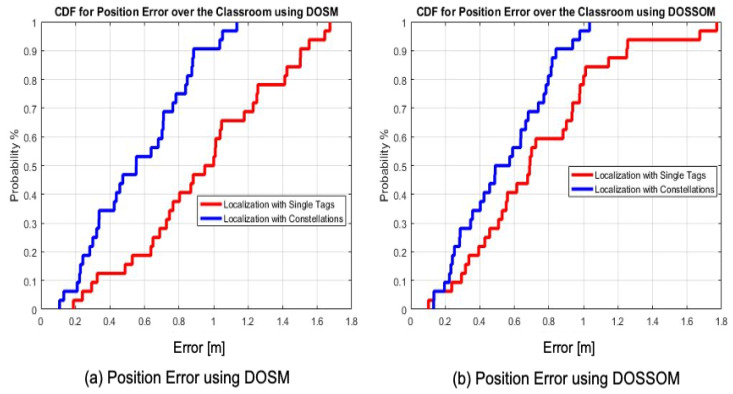
Cumulative Distribution Function (CDF) for the estimated position error using the multilateration technique for the classroom environment.

A reliable attenuation coefficient, corresponding to the classroom environment, was determined in the offline stage (Figure 12a). In the online stage, localization based on a single tag is performed with four independent RFID tags (Figure 13a). Each one is fixed on the center of each wall as shown in Figure 12b. The localization based on the constellation of tags was carried out with four constellations of four RFID tags each (Figure 13b). The center of each constellation is situated on the center of each wall. By applying the multilateration technique and following both empirical the DOSM and DOSSOM indoor propagation models, position errors were estimated at the thirty-two different positions illustrated in Figure 12b. They are presented through comparative cumulative distribution functions illustrated in Figure 14.

Taking into account the gathered results, the cumulative distribution function for the estimated positions’ errors at 90%, for the constellation of tags over the classroom environment, is equal to 88 cm and 81 cm with the DOSM and DOSSOM, respectively (Figure 14a,b). The optimal estimated position error, at a probability of 90%, obtained with the single tag scenario is equal to 1.25 m with the DOSSOM (Figure 14b). Thus, the efficiency of using the constellations of tags for localization purposes in an indoor environment is well proved. 

## 5. Conclusions

This work has presented a novel positioning system based on the use of constellations of RFID tags in order to increase the localization accuracy. The approach consisted of replacing a single tag with a group of tags. Various radii and a different number of tags per constellation are studied in-depth. The constellation approach has the same concept as that of MISO communication systems. Based on the signal diversity, the mean estimated distance errors using the averaging technique for the received power of the constellation are widely elaborated. Based on the measurements and simulations results, the optimal constellation is constituted of four RFID tags and has a radius equal to the wavelength. The constellation performances, in terms of the distance error, are compared to those obtained with the single tag scenario.

In essence, localization is completed with both single tag and constellation of tags scenarios by applying the multilateration technique. As a result, the better estimated positioning error is achieved thanks to the constellation of tags and was around 81 cm with the cumulative distribution function at 90%.

More investigations are required to further analyze the constellation approach in different and more complex scenarios. Future research will focus on studying the proposed approach in other indoor environments such as a hall and corridor, as well as on implementing other combining algorithms for the received signals of the constellation.

## Figures and Tables

**Table 1:** The mean estimated distance errors for different constellations.

Constellation’s Radius		*λ*/8 8.75	*λ*/4 17.5	*λ*/2 35	3*λ*/4 52.5	*λ* 70
Track A90	Mean Distance Error [m]	2.24	2.09	2.31	2.05	1.94
	Standard deviation	1.58	1.03	1.43	1.31	1.26

**Table 2:** The mean estimated distance errors for different constellations.

Constellation Tags’ Number	3 Tags	4 Tags	5 Tags	5 Tags (Penta)
Mean Distance Error [m]	2.0426	1.9421	1.9181	1.9533
Standard Deviation	1.0669	1.2555	1.0519	1.3073

**Table 3:** The mean estimated distance errors based on real measurements.

		Single Tag	Constellation of Tags
Track A60	MDE [m]	0.99	0.82
	Standard deviation	0.59	0.49
Track A90	MDE [m]	2.4	1.98
	Standard deviation	1.64	1.26
Track A120	MDE [m]	0.96	0.73
	Standard deviation	1.12	0.46

**Table 4:** The mean estimated distance errors by simulation.

		Single Tag	Constellation of Tags
Track A60	MDE [m]	1.02	0.81
	Standard deviation	0.59	0.45
Track A90	MDE [m]	2.41	1.93
	Standard deviation	1.62	1.21
Track A120	MDE [m]	0.97	0.71
	Standard deviation	1.04	0.49
